# Prevalence and factors associated with intestinal parasites among food handlers of food and drinking establishments in Aksum Town, Northern Ethiopia

**DOI:** 10.1186/s12889-017-4831-5

**Published:** 2017-10-17

**Authors:** Dejen Gezehegn, Mebrahtu Abay, Desalegn Tetemke, Hiwet Zelalem, Hafte Teklay, Zeray Baraki, Girmay Medhin

**Affiliations:** 1 Department of Public Health, Aksum University, P. O. Box: 298, Aksum, Ethiopia; 2 Department of Public Health, Asela University, Adama, Ethiopia; 3 Department of Biomedical Sciences, Aksum University, Aksum, Ethiopia; 4 Department of Nursing, Aksum University Aksum, Aksum, Ethiopia; 5 Aklilu Lemma Institute of Pathology, Addis Ababa, Ethiopia

**Keywords:** Intestinal parasites, Prevalence, Food handlers, Food and drinking establishments, Aksum, Ethiopia

## Abstract

**Background:**

Various epidemiological studies indicate that the prevalence of intestinal parasites is high in developing countries and those parasites are major public health importance in Sub-Saharan Africa. Their distribution is mainly associated with poor personal hygiene, environmental sanitation and lack of access to clean water. This study was conducted to estimate the prevalence of intestinal parasitic infection and identify factors associated with intestinal parasitic infection among food handlers in the Aksum Town of Tigray Regional State, North Ethiopia.

**Methods:**

A cross-sectional study design was used among 400 randomly selected food handlers who were selected as respondents. Data were collected by face to face interviewer administered questionnaire supplemented with observational checklist. Fresh stool samples were collected from respondents and were examined microscopically for the presence of any of intestinal parasites using standard laboratory methods. Multivariable logistic regression model using Adjusted Odds Ratio (AOR) and 95% Confidence Interval (CI) was fitted to analyze the independent predictors of intestinal parasitic infections.

**Result:**

The mean age of the food handlers included in this study was 26.0 years. Of the total respondents, 72.5% were females, 63% have completed at least secondary school educational level. Five species of Intestinal Parasites (IPs) were identified. The overall prevalence of being infected with at least one intestinal parasite was 14.5%, 95% CI (11.3, 18.0). The odds of being positive for at least one intestinal parasitic infection was 12.3 times higher among food handlers who practice medical checkup every 9 months compared to those who have a medical checkup every 3 months. The odds of being positive for intestinal parasitic infection was 3 times higher among food handlers with no formal education compared to those who have a level of education secondary school and above. Food handlers who received food hygiene and safety training were 66% less likely to be positive for at least one intestinal parasitic infection as compared to those who did not receive training.

**Conclusion:**

Prevalence of parasitic infection among food handlers observed in the current study is relatively low but is still a public health importance. Number of medical checkup, training in food hygiene and safety, feedback from customers and level of education were significantly associated with reduced odds of being infected with parasitic infection. Hence, these factors should be focused by policy makers and implementers to further bring the prevalence below the level of public health importance.

**Electronic supplementary material:**

The online version of this article (10.1186/s12889-017-4831-5) contains supplementary material, which is available to authorized users.

## Background

Food-borne diseases are the commonest types of infectious diseases among the global burdens of diseases in human beings especially in developing countries. Food handlers can play a vital role in the transmission of these parasitic infections [1]. Though there are so many sources of food and drinking contamination methods, food handlers serve as the main ones [1, 2]. If food handlers practice poor hygienic behavior, they could be carriers of pathogens [2]. According to World Health Organization (WHO) estimate, nearly one-third of the population in developed countries are affected by intestinal parasitic infections; whereas in developing countries the estimate is around five times higher [3–5]. In Ethiopia, intestinal parasitic infections (IPIs) are usually related to so many factors that are associated with poverty, including poor socioeconomic condition, poor hygiene and sanitation practice, lack of safe and adequate water supply and climate change [6]. There are many parasite species that can cause IPIs. Out of which *Ascaris Lumbricoides* is the most prevalent parasite followed by *Trichuris Trichiura*, and *hookworm* respectively [7]. The commonest mode of transmission of parasites is ingestion of food or water contaminated with the infective stages of one more species of the parasites [8]. There are different factors that affect the prevalence and severity of IPIs. Food handlers’ health condition as well as hygiene and sanitation practices are the commonest determinants of food and drinking contaminations. Infections can also be acquired through contaminated unwashed fingers, insects, circulation of banknotes and via wind during dry conditions [9].

In developing countries, where there is a poor regulatory system for food hygiene, food handlers are appointed in food and drinking establishment centers without investigating their health status for the common IPIs [10]. Individuals without symptoms of parasitic infections can be considered as dangerous to the society because such food handlers routinely practice their jobs without giving due attention for the transmission of infections. As a result, intestinal parasites can be transmitted to consumers directly or indirectly through food, water, nails and fingers from food handlers [11, 12].

Recently, changing lifestyle, breakdown of joint family system and increase in number of working women as food handlers has led to consumption of ready to eat foods. Individuals may be able to satisfy their taste and nutrition needs, but pays little attention to hygiene and food safety [13].

Aksum Town is one of the places of smokeless industry owners in the Tigray region. Due to this, eating and drinking in food and drinking establishments, such as hotels, restaurants and snack bars are becoming common practices. Information on intestinal parasites and associated factors among food handlers in the study area is limited. Thus, this study was aimed at determining the prevalence of intestinal parasites and identifying associated factors among food handlers working in food and drinking establishments in the Town.

## Methods

### Study design and setting

A community based cross sectional study was carried out among food handlers working in food and drinking establishments in Aksum Town, Northern Ethiopia from 14, September upto 10, November, 2015. Aksum Town is located 1067 Kilometers North of Addis Ababa, which is the capital City of Ethiopia, with a total population of 60,676. There are a total of 24 hotels, 33 restaurants, 50 snack bars and juice houses in Aksum Town. During the study period there were 1500 food handlers working in these places. Of these workers, 640 were working in hotels, 525 in restaurants and the rest 335 in snack bar and juice houses serving as food handlers in the Town (Aksum Town Administration report of 2015).

### Source and study population

The source population was all food handlers working in food and drinking establishments in Aksum Town and the study population were all randomly selected food handlers working in the food and drinking establishments of the Town.

### Sample size determination and sampling procedure

The sample size was calculated using a single population proportion formula with the assumptions of 95% confidence interval and 5% of marginal error; **n =** (Ζ_**1-α/2**_ **)** ^**2**^ **P (1 – P) /d** ^**2**^ **,** where “**n**” is the required sample size. Taking *p* = 44.1% (0.441) (the prevalence of intestinal parasites among food handlers in Yebu Town, South West Ethiopia) [14], and adding 5% non-response rate, a total of 400 food handlers participated in the study. Simple random sampling technique using lottery method was employed to reach the respondents from the roster lists of food handlers’ which obtained from the food and drinking establishments.

### Variables and measurement

The dependent variable is a condition of intestinal parasitic infection (infected versus uninfected), and the independent variables include socio-demographic, individual, economic and food handling practice related variables.

*Food handlers with IP*: those who had one or more detected intestinal parasites, such as (*E. histolytica, G. lamblia, Hookworm, A. Lumbricoides, T. Trichiura, H. nana and S. mansoni*), using microscopic examination.

Knowledge of food handlers on IP: food handlers who scored above the mean value of the 13 knowledge questions was considered as having good knowledge (Additional file 1).

### Data collectors and data collection procedures

A structured questionnaire which was adopted from WHO food checklist [*https://www.ces.ncsu.edu/wp-content/uploads/2014/03/SOPcklistHACCP.pdf* *]* WHO food preparation manual [http://www.who.int/foodsafety/publications/consumer/manual_keys.pdf], and other literatures were used to interview the selected food handlers. The reliability of knowledge questions was measured using Cronbach’s alpha and the result was 0.80. First, the questionnaire was written in English and then translated to the local language, *Tigrigna* and back translated to English to ensure its consistency. The *Tigrigna* version of the questionnaire was pretested among 15 food handlers in Adwa Town. Data related to socio-demographic characteristics and personal hygiene practices were collected via face to face interview method. Three environmental health and two medical laboratory professionals were recruited for data collection, supervision and microbiological analysis. The data collectors were trained for 2 days by the principal investigator on observational data collection and specimen collection procedures. After interviewing, respondents were asked to give a fresh stool specimen in a sterile, clean wide-mouthed plastic container by clean wooden applicators stick for microbiological analysis. Fresh stool samples were taken from all the 400 respondents.

Intestinal parasites were investigated microscopically from each stool sample using both direct smears mount in saline and formal-ether concentration sedimentation procedures as per the standards [15]. Specimens were preserved using stool preservatives such as polyvinyl alcohol (PVA) and 10% formalin in case of delay. The stool transported to laboratory within an hour of collection using stool cup. Code was given for each stool specimen sample collected from the food handlers on the outer face of the plastic cup for possible treatment of positive findings after examination. Saline wet mount for the common parasites, and concentration and floatation technique for the parasites which are found in stool in small numbers has been used. Kato Katz techniques have also been used for parasites which are rarely found in stool especially *Schistosoma* species.

### Data quality assurance mechanisms

Experienced lab technicians, with at least 2 years’ work experience, was recruited for laboratory examinations. Before the actual stool specimen’s examination, pre-test was conducted on five stool samples collected from patients visiting a health center to look the reliability or reproducibility of the stool examination procedure by the laboratory technicians with two different instruments/microscopes. A close supervision was applied during stool sample collection to make sure the participants bring their own stool specimen. Cleaning, coding and entering of the data was carried out carefully. A standard operational procedure of examinations was followed, slides were blindly cross checked and rechecked by other laboratory technology experts, standardization of equipment (microscopes, slides and chemicals) were used and full field of the stool were examined in order not to miss parasites in each stool examination.

### Data processing and analyses

Data were entered into Epi-info version 7.0 software package and transferred to SPSS version 21 software for analysis. Frequencies and percentages were generated for all variables in this study. Odds ratios (both crude and adjusted) with corresponding 95% confidence intervals were used to assess the strength of associations. Independent variables resulting with a *p*-value of less than 0.2 on bivariable analysis were considered in the multivariable logistic regression analysis. The goodness of fit model was checked by Hosmer Lemeshow statistic and p-value greater than 0.05 was considered as a fit model. Summary of the findings is presented in tables and described by narration.

## Results

### Socio-demographic characteristics of respondents

A total of 400 food handlers aged 18–50 years who had been working in food and drinking establishments were interviewed with a 100% response rate. The mean age of the respondents was 26.04 (Standard Deviation = 6.31) years. Among the total respondents, the majority, 389 (97.25%) were Orthodox Christian and few of them, 68 (17%) were unable to read and write. Most of them, 339 (84.8%) have been serving for a period of less than 5 years in food and drinking establishments. Their monthly income was less than < 30 USD for about 162 (40.5%) of the respondents (Table 1).

**Table 1:** Socio demographic characteristics of food handlers in Aksum Town, Northern Ethiopia. (Sep-Nov 2015), (*n* = 400)

Variables		Diagnosis for at least one parasite				P-value
		Negative		Positive		
		Number	Percent	Number	Percent	
Age	18–20 years	64	18.7	11	19.0	0.400
	21–30 years	237	69.3	36	62.1	
	31–40 years	31	9.1	9	15.5	
	> 40 years	10	2.9	2	3.4	
Sex	Female	245	71.6	45	77.6	0.300
	Male	97	28.4	13	22.4	
Religion	Orthodox	331	96.8	58	100.0	0.100
	Muslim	11	3.2	0	0.0	
Education	Illiterate	46	13.45	22	38.00	≤ 0.001
	Primary school	70	20.46	10	17.24	
	Secondary school or above	226	66.08	26	44.82	
Monthly income	< 30 USD	129	37.7	33	56.9	0.030
	[30–65) USD	181	52.9	23	39.7	
	[65–109) USD	28	8.2	2	3.4	
	≥ 109 USD	4	1.2	0	0.0	
Service year	< 1 year	68	19.9	14	24.1	0.600
	1–5 year	223	65.2	34	58.6	
	6–10 year	51	14.9	10	17.2	

### Personal hygiene related factors of food handlers

Of the total respondents, 310 (77.5%) reported that they always wash their hands before food preparation, 295 (73.8%) always use soap and water after visiting toilet and 303 (75.8%) wash their hands after touching dirty material and different body parts. Finding from observation also supported that personal hygiene was practiced by the food handlers.

Similarly, 286 (71.5%) wash their body regularly in their working area, and 354 (88.5%) have medical certificate which they renewed every 3 months (*n* = 279; 69.8%), every 6 months (*n* = 59; 14.8%) and every 9 months (*n* = 19; 4.8%). Linked to their personal hygiene, 89 (22.3%), 152 (38%) and 131 (32.8%) cut their finger nail twice a week, once a week and once in 2 weeks, respectively. Only 28 (7%) of respondents said no need to cut their nails regularly. Bivariate analysis of the personal hygiene factors indicates that food handlers having poor personal hygiene practice were more exposed for intestinal parasitic infection (Table 2).

**Table 2:** Intestinal parasitic infection and personal hygiene practice of food handlers in Aksum Town, Northern Ethiopia. (Sep -Nov 2015), (*n* = 400)

Variables		Diagnosis result of intestinal parasites			
		Negative		Positive	
Hand wash before food		Number	%	Number	%
Preparation	Always	277	81.0	33	56.9
	Usually	51	14.9	14	24.1
	Sometimes	14	4.1	11	19.0
Hand wash by soap and water after visiting toilet	Always use soap and water	265	77.5	30	51.7
	Usually use soap and water	50	14.6	11	19.0
	Sometime use soap and water	27	7.9	17	29.3
Hand wash after touching dirty materials	No	69	20.2	28	48.3
	Yes	273	79.8	30	51.7
Wash your body regularly in the working area	No	75	22.7	18	36.7
	Yes	255	77.3	31	63.3
Medical certificate	No	37	10.8	9	15.5
	Yes	305	89.2	49	84.5
Medical checkup	Every 3 months	255	83.1	24	48.0
	Every 6 months	44	14.3	15	30.0
	Every 9 months	8	2.6	11	22.0
Wear clean aprons when preparing food	No	42	12.3	15	25.9
	Yes	300	87.7	43	74.1
Wear hair garments during food preparation	No	74	21.6	20	34.5
	Yes	268	78.4	38	65.5
How frequent do you cut finger nail	Twice a week	83	24.3	6	10.3
	Once a week	134	39.2	18	31.0
	Once in two weeks	100	29.2	31	53.4
	No need to cut	25	7.3	3	5.2

### Knowledge and working area related factors of food handlers

A little over half (*n* = 174; 50.9%) of the respondents had a good level of knowledge about transmission and prevention mechanisms of intestinal parasites. The majority, 342 (85.5%) were users of private tap water, 373 (93.3%) had shower facility and all of the respondents had a toilet facility in their establishment. Of the establishments which had toilet facility, 364 (91%) had water flush type of toilet. Only 176 (44%) respondents were trained about food hygiene and safety (Table 3).

**Table 3:** Intestinal parasitic infection with respect to knowledge and working area related factors of food handlers in Aksum Town, Northern Ethiopia. (Sep -Nov 2015), (*n* = 400)

Variables		Diagnosis result of intestinal parasites			
		Negative		Positive	
		Number	%	Number	%
Sources of water in your working area	Protected hand dug well	40	11.7	18	31.0
	Private tap water	302	88.3	40	69.0
Working are have shower facility	No	18	5.3	9	15.5
	Yes	324	94.7	49	84.5
Working area have separate dressing	No	80	23.4	14	24.1
Room	Yes	262	76.6	44	75.9
To clean utensils and drinking cup	Water and detergent	190	55.6	41	70.7
	Hot water and detergent	69	20.2	4	6.9
	Water with bleach	83	24.3	13	22.4
How frequently is the kitchen floor Cleaned	Once a day	129	37.7	27	46.6
	Twice a day	111	32.5	20	34.5
	Three times a day	102	29.8	11	18.9
Toilet facility of working area	No	0	0.0	0	0.0
	Yes	342	100.0	58	100
Type of toilet	Water flesh	310	90.6	54	93.1
	VIP latrine	32	9.4	4	6.9
Have you ever received feedback	No	81	23.7	25	43.1
	Yes	261	76.3	33	56.9
Supervise by owner or manager	No	42	12.3	8	13.8
	Yes	300	87.7	50	86.2
Food hygiene and safety training	No	176	51.5	48	82.8
	Yes	166	48.5	10	17.2
Knowledge on IP	Poor knowledge	168	49.1	35	60.3
	Good knowledge	174	50.9	23	39.7

*IP* Intestinal Parasites

### Prevalence and types of intestinal parasite

Based on microscopic stool sample examinations, five species of intestinal parasites were identified with an overall prevalence of 14.5%, 95%CI (11.3, 18.0). *G. lamblia*, identified in 21 (5%) of the respondents, was the most prevalent parasite followed by *E. histolytica, S. mansoni*, *H. nana* and *Hookworm* with prevalence of 14 (3.3%), 10 (2.5%) and 4 (1%), respectively. Double infection of *E. histolytica* and *G. lamblia* as observed only on one respondent. Among the respondents with IPs, 35 (60%) were from restaurants, 10 (17%) from hotels and 13 (22.4%) from café and juice houses (Table 4).

**Table 4:** Prevalence of intestinal parasites detected from stool specimens of food handlers in Aksum Town, Northern Ethiopia. (Sep -Nov 2015). (*n* = 400)

Parasites	Number positive	Percent positive
*Giardia Lamblia*	20	5.0
*Entamoeba Histolytica*	13	3.3
*Schistosoma Mansoni*	10	2.5
*Hymenolepis Nana*	10	2.5
*Hookworm*	4	1.0
*Giardia Lamblia and Entamoeba Histolytica*	1	0.3

### Factors associated with intestinal parasitosis among the food handlers

From the bivariate analysis 15 variables met the criteria (*p*-value < 0.2) to select variables for multivariable analysis. Multi-collinearity of these variables was near to one tolerance and VIF was < 10. Among those 15 variables, four variables (frequency of medical checkup, training on food hygiene and safety, feedback from customer and level of education) were significantly and positively associated with parasitic infection (*P*-value < 0.05).

In a multivariable logistic regression analysis, food handler who practiced medical checkup every 9 months were 12.3 times more likely to be positive for intestinal parasites compared to those who practice medical checkup every 3 months (AOR = 12.33, 95% CI = 3.23–46.76). Similarly, the odds of being positive for intestinal parasitic infection was 3 times higher among food handlers who did not attend formal education compared to those who were secondary school and above (AOR = 3.01, 95% CI = 1.03–8.20). Food handlers who received feedback or advice from their customers about hygienic practice were 64% less likely to be positive for intestinal parasites compared to those who did not receive feedback (AOR = 0.36; 95% CI = 0.15–0.89). Food handlers who received food hygiene and safety training were 66% less likely to be positive for intestinal parasites compared to those who did not receive training (AOR = 0.34; 95% CI = 0.12–0.96) (Table 5).

**Table 5:** Multivariable logistic regression analysis of predicators for intestinal parasitic infection among food handlers in Aksum Town, Northern Ethiopia. (Sep -Nov 2015), (*n* = 400)

Variables	Positive for intestinal parasite			COR (95% CI)	*P*-value	AOR (95% CI)
		Yes	No			
Education level	Illiterate	22	46	4.15(2.17–7.96)	< 0.001	3.0(1.03–8.20)*
	Primary school	10	70	1.24(0.57–2.70)	0.481	0.70(0.24–2.05)
	Secondary school and above	26	226	1		1
Source of water	Protected hand dug well	18	40	3.39(1.78–6.48)	< 0.001	2.48(0.85–7.19)
	Private tap water	40	302	1		1
Hand washing before food preparation	Always	33	277	1		1
	Usually	14	51	2.30(1.15–4.60)	0.018	0.98(0.23–4.18)
	Sometimes	11	14	6.59(2.76–15.71)	< 0.001	2.21(0.43–11.31)
Hand washing by soap and water	Always use it	30	265	1		1
	Usually use it	11	50	1.94(0.91–4.13)	0.084	1.04(0.30–3.51)
	Sometime use it	17	27	5.56(2.72–11.36)	< 0.001	1.82(0.36–9.20)
Hand washing after touching dirty material	No	28	69	3.69(2.07–6.58)	< 0.001	1.72(0.68–4.33)
	Yes	30	273	1		1
Medical checkup for IP	Every 3 months	24	255	1		1
	Every 6 months	15	44	3.62(1.76–7.44)	< 0.001	2.36(0.89–6.25)
	Every 9 months	11	8	14.60(5.36–39.80)	< 0.001	12.33(3.23–46.76)*
How frequent the kitchen floor cleaned	Once a day	27	129	1		1
	Twice a day	20	111	0.86(0.45–1.61)	0.642	1.40(0.52–3.80)
	Three times a day	11	102	0.51(0.24–1.08)	0.082	1.40(0.44–4.34)
Received feedback from customers	No	25	81	1		1
	Yes	33	261	0.40(0.23–0.72)	0.002	0.36(0.15–0.89)*
Received food hygiene training	No	48	176	1		1
	Yes	10	166	0.22(0.10–0.45)		0.34(0.12–0.96)*
Knowledge of food handlers on IPs	Poor knowledge	35	168	1.576(.894–2.779)	0.116	0.82(0.33–2.05)
	Good knowledge	23	174	1		1

*significant at p-value *< 0.05, COR* Crude Odds Ratio, *AOR Adjusted Odds Ratio, IP* Intestinal Parasites

## Discussion

The prevalence of parasitic infection reported in the current study is consistent with a study done among bakery workers in Iran (11.9%) [16] and is relatively higher than the 6.9% reported in Khartoum Sudan [11]. But it is relatively lower as compared to the findings of other countries which reported 29.3% in India [17], 23.0% in Saudi Arabia [18], 23.7% in Kenya [19], 21.6% in Ghana [20]. It is also much lower than 41.1% reported in Bahirdar [21], 44.1% in Yebu, 45.3% in Addis Ababa [22] and 49.3% in Mekelle [23]. Such a relatively high prevalence of intestinal parasites is largely due to variation in year of study, socio-demographic characteristics, personal hygiene practice and environmental sanitation, safe water supply, health promotion practice, food hygiene and safety training, knowledge of transmission and prevention of intestinal parasite differences.

The predominant parasite identified in the present study was *G. lamblia* with a prevalence of 5%, which is relatively consistent with similar studies from Bakery workers in Iran and Saudi Arabia reports prevalence of 3.7% [16] and 9% [18] respectively. The predominance of this parasite could be due to the easy mode of transmission of the parasite which is usually found in food, water, soil or contaminated surface with the feces.

In the present study, the rate of contamination with intestinal protozoa, 34(8.55%) is higher than that of intestinal worms, only 4(1%) which is in line with studies from Addis Ababa University student cafeteria, Ethiopia 86 (50%) and 2 (1.16%) [24] and Saudi Arabia 27 (13.5%) & 1 (0.5%) [18], respectively. This could be reasoned out by the transmission and distribution of protozoa through cysts is more direct and simpler than worms which need special consideration.

The finding of this study showed that 69.80%, 14.5% and 4.80% of the food handlers had taken medical checkup every 3 months, every 6 months and every 9 months, respectively. This figure is incongruent to a report from Mekelle, Ethiopia which is 63.2% (examined every 6 months) [23] and from Jimma Town, Ethiopia which is 56.7% (examined every 6 months) [25]. But a study conducted in Bahirdar Town, Ethiopia reported that none of the respondents came across regular medical examinations[21]. This gap could be due to differences in level of education, regular supervision by regulatory team and time schedule of renewing medical checkup. Currently, the ministry of health recommends food handlers to renew their medical certificate every 3 months.

Food handlers who received feedback from customers were 64% less likely to be positive for intestinal parasite compared to those who did not. This was supported by a result from Gondar where respondents who receive feedback from their customers on how to control and prevent intestinal parasite infection and transmission [26]. This might be due to the fact that those food handlers receiving feedback would have more tendency to apply the prevention methods of IPs.

The present study showed that 44% of the respondents were trained on food handling and safety. This finding is greater than the study conducted in Bahirdar Town, Ethiopia (14%) [21] and supported by the findings of other similar studies [23, 26]. This might be because there is difference in number of institutions working in safety, tendency of employers to recruit food handlers without considering health certificate as a basic criterion and low monthly salary (payment) for food handlers in the other study areas [27].

In the current study, the odds of being positive for intestinal parasitic infection was 3 times higher among food handlers who were not formally educated compared to those who were secondary school and above. This is to mean that food handlers who had a low level of knowledge about transmission and prevention of IPs had a higher chance of being infected by IPs compared to those who had better knowledge as a result of their formal secondary school education and above. Most of the food handlers knew how to keep food safely, but they do not put this into practice when they are observed, which is also supported by other studies where food handlers did not usually translate their knowledge into practice [28].

## Conclusion and recommendations

In conclusion, compared to other similar studies conducted in developed and developing countries, relatively lower prevalence 56 (14.0%) food handlers were tested positive for different intestinal parasites. Of the number of variables analyzed, frequency of medical checkup, food hygiene and safety training, feedback from customers and level of education were the identified factors affecting food handlers to intestinal parasites in the study area.

Employers, managers or owners of food and drinking establishments, should continuously supervise the food handlers and establish personal hygiene rule and posted it on easily visible site. Environmental health practitioners should strongly continue conducting periodic inspection, design and implement food safety awareness creation program, establish rules on periodic medical examination and implement and prepare food hygiene training manual, guideline and a certificate.

## Additional files

Additional file 1: Knowledge question. (DOCX 14 kb)
