# Supplementary material for: Prevalence and factors associated with intestinal parasites among food handlers of food and drinking establishments in Aksum Town, Northern Ethiopia
Source: BMC Public Health. 2017 Oct 17;17:819. doi: 10.1186/s12889-017-4831-5 (PMC5645889; doi:10.1186/s12889-017-4831-5)
Supplement: Supplementary file 1 — Knowledge question. (DOCX 14 kb) [file 12889_2017_4831_MOESM1_ESM.docx]

| **Knowledge related questions about intestinal parasites and their transmission.** | | |
| --- | --- | --- |
| 30 | Have you ever heard about intestinal parasites? | 1. Yes 2. No |
| 31 | Do you know the sources of intestinal parasites? | 1. Yes 2. No |
| 32 | If your answer is yes, what are the sources of intestinal parasites? | 1. Contaminated food 2. Contaminated water 3. Sexual contact 4. Other specify________ |
| 33 | Can intestinal parasites be transmitted? | 1. Yes 2. No |
| 34 | If yes, how could it be transmitted? | 1. Direct contact 2. Fecal contamination 3. Sexual contact 4. Other specify________ |
| 35 | Can some intestinal parasites be transmitted during bare foot movement? | 1. Yes 2. No |
| 36 | If your answer is yes which parasite? | Specify___________ |
| 37 | Can some intestinal parasites be transmitted during swimming? | 1. Yes 2. No |
| 38 | If your answer is yes which parasite? | Specify___________ |
| 39 | Can intestinal parasites preventable? | 1. Yes 2. No |
| 40 | If your answer is yes, do you know how intestinal parasites could be prevented? | 1. Personal hygiene 2. Drinking alcohol 3. Environmental sanitation 4. Food hygiene 5. Eating cooked meat and vegetables 6. Other specify________ |
| 41 | Do they have treatment? | 1. Yes 2. No |
| 42 | Do you think intestinal parasites are fatal disease? | 1. Yes 2. No |
